# Epigenome-Wide DNA Methylation Profiling of Matched Primary Breast Cancers and Brain Metastases Reveals Epigenetic Markers linked to Metastatic Progression: A Preliminary Study

**DOI:** 10.7150/ijms.130407

**Published:** 2026-10-02

**Authors:** Beate Alinger-Scharinger, Georg Hutarew, Christoph Schwartz, Alexander Romagna, Ognian Kalev, Serge Weis, Simon P. Gampenrieder, Gabriel Rinnerthaler, Vanessa Castagnaviz, Franz Gassner, Roland Geisberger, Nadja Zaborsky, Richard Greil, Karl Sotlar, Theo F. J. Kraus

**Affiliations:** 1 Institute of Pathology, University Hospital Salzburg, Paracelsus Medical University, Müllner Hauptstr. 48, A-5020 Salzburg, Austria.; 2 Department of Neurosurgery, University Hospital Salzburg, Paracelsus Medical University, Salzburg, Austria.; 3 Department of Neurosurgery, München Klinik Bogenhausen, Munich, Germany.; 4 Division of Neuropathology, Neuromed Campus, Institute of Pathology and Molecular Pathology, Kepler University Hospital, Johannes Kepler University, Linz, Austria and Clinical Research Institute for Neurosciences, Johannes Kepler University Linz, Austria.; 5 Clinic of Internal Medicine 3, University Hospital Salzburg, Paracelsus Medical University, Salzburg, Austria.; 6 Salzburg Cancer Research Institute - Laboratory for Immunological and Molecular Cancer Research (SCRI-LIMCR), Salzburg, Austria.; 7 Cancer Cluster Salzburg, Salzburg, Austria.; 8 Division of Oncology, Department for Internal Medicine, Medical University Graz, Graz, Austria.; 9 Department of Neurosurgery, Medical University of Innsbruck, Innsbruck, Austria.

**Keywords:** breast cancer, brain metastases, metastasis, methylome, epigenetic profiling

## Abstract

**Introduction:**

Breast cancer represents the most prevalent malignancy among women globally, with a rising incidence of metastases to the central nervous system. The presence of brain metastases is associated with a poor prognosis, largely due to the limited availability of effective treatment modalities.

**Methods:**

In this preliminary study, we performed an epigenome-wide DNA-methylation analysis of matched primary breast cancer and brain metastasis of the same patients applying the Illumina Infinium EPIC Bead Chip interrogating more than 850,000 CpG sites at single base resolution.

**Results:**

We found that primary breast cancers and corresponding metastases are very similar on the global DNA methylation level. Detailed analysis showed aberrant methylation patterns that reflect the process of brain metastasis. We identified 107 nominally significant methylated CpG sites (DMCGs) within promoter and gene regions (Δβ ≥ 0.1, *p*-value < 0.05), when comparing primary breast tumors to their brain metastatic counterparts. However, due to the limited sample size, no individual CpG site retained statistically significant after correction for multiple testing.

**Conclusion:**

Our study highlights specific aberrant DNA methylation patterns that are associated with brain metastasis in breast cancer. Characterizing these epigenetic alterations provides a promising avenue for identifying novel therapeutic targets and clinically relevant biomarkers.

## Introduction

Metastatic breast cancer (MBC) represents the second most common malignancy associated with the development of brain metastases (BM), following lung cancer 1-5. Breast cancer brain metastases (BCBM) are clinically correlated with poor prognosis, frequently accompanied by neurological impairments that adversely affect both life expectancy and quality of life. Although direct spread from breast cancer (BC) to BM is not uncommon, BM typically arises as a late-stage event in the course of disease progression, often following metastases to the lung, liver, and/or bone, with bone being the most prevalent site of BC dissemination.

The increasing incidence of BCBM is likely attributable to advancements in systemic therapies, which have extended the survival of patients with MBC 6. Specific BC subtypes, most notably human epidermal growth factor receptor 2 (HER2)-positive and triple-negative breast cancer (TNBC; hormone receptor [HR]-negative/HER2-negative), exhibit an elevated risk of BCBM development 7. Furthermore, younger age at BC diagnosis, negative estrogen receptor (ER) status, and high expression of the proliferation marker Ki-67 have been significantly associated with increased susceptibility to BCBM 3.

The formation of BCBM involves a complex, multistep biological cascade, including epithelial-mesenchymal transition (EMT) and translocation of tumor cells across the blood-brain barrier (BBB) into the central nervous system. The molecular and cellular mechanisms governing these processes have been extensively characterized 2. Of particular interest is the interaction between disseminated tumor cells, whether originating from primary or metastatic brain lesions, and the brain tumor microenvironment, which facilitates colonization and proliferation 8, 9.

While therapeutic strategies for primary breast tumors have improved considerably, the onset of brain metastases remains associated with poor clinical outcomes. This limitation is largely due to the restrictive nature of the BBB, which impedes the effective penetration of most targeted therapies into the central nervous system 10, 11. Encouraging results from recent investigations into anti-HER2 therapies for BCBM have been reported 12, 13. However, such systemic treatments tailored to specific BC subtypes face constraints, as genomic profiling has revealed significant biological differences between primary breast cancers (PBC) and BCBMs, including the loss of hormone receptor expression, alterations in HER2 status, and dysregulation of PI3K signaling pathways 14-16. Additionally, profound neurological symptoms often hinder the administration of anticancer agents and diminish patients' quality of life.

Epigenetic regulation plays a pivotal role in fundamental biological processes such as cellular differentiation, imprinting, and gene activation or silencing. Similar to genetic mutations, epigenetic dysfunction can lead to disease, even in the absence of changes to the underlying DNA sequence. Aberrant epigenetic modifications are increasingly recognized as key contributors to tumorigenesis and metastatic progression. Therefore, investigating epigenetic alterations may offer novel insights for improving screening approaches for both primary BC and BCBM.

In this preliminary study, we conducted integrated, epigenome-wide DNA methylation profiling on highly selective tissue samples derived from matched pairs of primary breast cancers and corresponding brain metastases. Using the Illumina EPIC BeadChip array, we identified distinct methylation signatures in genomic pathways, highlighting epigenetic modifications associated with metastatic transition.

## Methods

### Tissue collection

We analyzed 20 anonymized tissue samples obtained from ten patients diagnosed with metastatic breast cancer. For each patient, paired samples were collected, comprising both primary breast tumor tissue and the corresponding brain metastasis tissue. Formalin-fixed, paraffin-embedded (FFPE) specimens, which had undergone routine histopathological diagnostic procedures, were retrieved from the archives of the Institute of Pathology and Molecular Pathology, Kepler University Hospital, Johannes Kepler University Linz, and the University Institute of Pathology, University Hospital Salzburg, Paracelsus Medical University.

This study was conducted in compliance with the principles outlined in the Declaration of Helsinki and received approval from the Ethics Committee of Salzburg (reference number: 415-E/2262/4-2018). Informed consent was obtained from all participants and/or their legal guardians prior to inclusion.

Inclusion criteria were the confirmed diagnosis of brain metastasis through standard diagnostic procedures and enough tumor material for isolation of at least 500ng of DNA both from primary BC and BCBM. No restrictions were applied regarding patient age, tumor classification, histological grading, tumor size, lymph node involvement, or the status of estrogen receptor, progesterone receptor, HER2, or Ki-67 expression in a representative primary breast cancer (Figure 1A-E) and the corresponding brain metastasis (Figure 1F-J). All tissue samples were pseudonymized before analysis to ensure non-identifiability. Detailed clinical and pathological characteristics of the patient cohort are presented in Supplementary Table S1.

### Molecular genetic analysis

Molecular genetic analyses were performed as previously described 17-21. Representative viable tumor tissue was microscopically identified and dissected. DNA extraction was performed applying the Maxwell system (Promega) according to the manufacturer's protocol. Methylation profiling was performed using the Infinium Methylation EPIC Bead Chip (Illumina) protocol according to the manufacturer's protocol 17-19. The DNA input amount for bisulfite conversion was 500ng as standardized and accredited in our lab. Arrays were scanned on an Illumina NextSeq 550 DX device. Raw intensity data files (idat-files) were subjected to downstream analysis.

### Computational data analysis

Methylation data were processed using the Illumina Genome Studio Methylation Module (2011.1) and RnBeads (version 2.4.0) 22 as previously described 23. Methylation calls were transferred to beta values in the range from 0 to 1 (i.e. 0% of methylation to 100% of methylation) 22. Probes were annotated to the four genomic regions tiling (genome tiling regions of length 5000), genes (version Ensemble genes 75), promoters (version Ensemble genes 75), and CpG islands (CpG island track of UCSC Genome Browser) 22. We used “limma” for testing mean methylation differences 22. Nominal p-values were applied as performed in other epigenome-wide association studies (EWAS) 24, 25. To minimize technical bias, samples from different experimental groups were randomly distributed across processing batches and microarrays (Sentrix-ID/array barcode and the processing date can be found in Supplementary Table S1). Further analyses were performed using Morpheus and GraphPad Prism software suite (version 9).

## Results

### Patient characteristics

In the course of our study, we analyzed a well-defined but very heterogeneous collective of 20 anonymized tissue samples obtained from ten patients diagnosed with MBC (Supplementary Table S1). Patients' age ranged from 37-62 years, except one case that showed lobular histology all others were classified as no special type (NST) and immunohistochemical (IHC) subtypes varied between TNBC (4/10), HER2 positive (2/10) and HER2 negative (4/10) BC, respectively. The receptor status changed in 4/10 patients between primary BC and BCBM. Histological grade was G2 (4/10) and G3 (6/10) in primary BC samples and high histologic grade (G3) in all of the BCBM (10/10). The tumor cell content within the samples varied from a minimum of 50% to a maximum of 95% of tumor cells and the identified stromal TILs (tumor infiltrating lymphocytes) within the tissue samples showed a distribution from 1% to 40%.

### Primary breast cancer and brain metastasis show high similarities on the global DNA methylation level

Methylation analysis of 20 samples from ten patients with each primary breast cancer and brain metastasis was performed. Applying the Illumina Infinium EPIC bead chip, we interrogated 866,895 methylation sensitive probes per sample. During preprocessing, we removed 17,371 probes that overlapped with SNPs, 79,261 probes applying greedycut filtering, 2,124 context specific probes and 17,009 probes on sex-chromosomes; this resulted in a total of 751,130 retained valid methylation probes (Figure 1K). An analysis of global beta value distributions showed high densities with beta values <0.1 and >0.8 (Figure 1L).

To assess the similarity between analyzed samples, we performed a principal component analysis (PCA). Focusing on methylation profiles at CpG islands, the PCA did not reveal two clearly distinct clusters separating primary breast cancer from corresponding brain metastases:

Unexpectedly, individual pairs of primary tumors and their matched brain metastases exhibited close spatial proximity within the PCA plot, indicating conserved epigenetic signatures between the two sample types (Figure 2A). This result suggests that, despite the biological transition associated with metastasis, the epigenetic landscape at promoter regions may retain subtype-specific patterns reflective of the primary tumor. Such observations could hold relevance for biomarker development and understanding the mechanisms underlying brain colonization.

Subsequently, we carried out unsupervised hierarchical clustering leveraging a random forest algorithm applied to the top 1,000 most variable CpG sites. This analysis aimed to further interrogate methylation-based relationships between primary breast tumors and their matched brain metastases. Consistent with our principal component analysis findings, the clustering did not result in the emergence of two clearly distinct groups separating the primary tumors from the metastatic counterparts. Instead, samples from individual patients, i.e. those of the primary breast cancer and its corresponding brain metastasis, were frequently found in close proximity within the resulting dendrogram (CpG islands with the dissimilarity metric correlation based and the agglomeration strategy average) (Figure 2B). This spatial clustering suggests a preservation of epigenetic features during metastatic progression to the brain and reinforces the notion of patient-specific methylation patterns.

Together, these findings may reflect the evolutionary continuity between primary and metastatic disease within the same individual, and underscore the potential utility of DNA methylation signatures in tracking tumor origin and progression.

### Primary breast cancer and the corresponding brain metastasis show distinct differences in methylation signatures

Differential DNA methylation analysis was conducted to compare primary breast cancer tissue with corresponding brain metastases. To identify significantly altered methylation sites, we performed pairwise comparisons using a threshold of Δβ ≥ 0.1 and p-value < 0.05. In the resulting volcano plot, probes meeting these criteria are highlighted in red (Figure 2C).

Subsequently, we generated a scatter plot to visualize methylation differences between primary breast cancer and brain metastasis tissue, emphasizing the top 100 regions ranked by combined statistical relevance (Figure 2D).

The analysis revealed a total of 107 nominally significantly methylated CpG sites (DMCGs) located within gene and promoter regions, comprising 97 within promoter regions and 10 within gene bodies, based on the defined statistical thresholds (Δβ ≥ 0.1; p-value < 0.05). Of note, the list of top ten nominal significantly methylated genes and promoters include *HMGB1P7, DEFB1, MIR5089, NKX2-6, OR51A10P, LINC00528, SLC15A3, PCDHGB7, MIR4708,* and* RNU6-993P* (Figure 3A, Supplementary Table S2). Methylation levels show significant differences in primary breast cancer compared with brain metastasis. The gene *DEFB1* shows a hypomethylation in BCBM compared to primary BC whereas *LINC00528* (long non-coding RNAs) shows significant hypermethylation in BCBM. The hypomethylated promoter regions of BCBM compared to BC comprise *HMGB1P7, MIR5089* (miRNAs) and *OR51A10P* contrary to the promoters of *NKX2-6, SLC15A3, PCDHGB7, MIR4708* and *RNU6-993P* which showed hypermethylation in BCBM.

### *In silico* screening of core loci for therapeutic potential under redox perturbation

To evaluate the translational and therapeutic potential of the identified differentially methylated regions, we cross-referenced our top loci with the specialized breast cancer multi-omics database CAPmed-BC. 68, 69 We focused on the three successfully mapped core genes: *DEFB1, PCDHGB7*, and *SLC15A3* (Figure 3B).

The transcriptomic profiles under cold atmospheric plasma (CAP)-induced redox stress revealed distinct, subtype-specific regulation mechanisms. For *DEFB1*, expression levels were virtually zero across nearly all conditions in both non-TNBC and TNBC models, indicating robust baseline transcriptional silencing.

In contrast, *PCDHGB7* displayed dynamic, time-dependent responsiveness. In non-TNBC cells, *PCDHGB7* expression transiently dropped one hour post-treatment before undergoing a compensatory upregulation at eight hours. Conversely, the aggressive TNBC subtype showed a highly suppressed baseline expression of *PCDHGB7*, which was transiently alleviated by a multi-fold transcriptional induction one hour after redox perturbation.

Similarly, *SLC15A3* exhibited a heavily repressed baseline state, particularly in TNBC cells where expression was mostly absent. However, redox stress triggered a subtle, stochastic activation of *SLC15A3* in specific TNBC replicates after both one and eight hours.

Taking together, these *in silico* validation data demonstrate that the identified epigenetically altered loci are transcriptionally reactive to therapeutic oxidative stress challenges.

### Enrichment analysis of differentially methylated CpGs (DMCGs) reveals distinct altered pathways in primary breast cancer and brain metastasis

Gene Ontology (GO) enrichment analysis was conducted to identify functionally enriched biological processes associated with differentially methylated genes (Figure 3C, D). In brain metastases compared with primary breast cancer tissue, hypermethylated genes were predominantly associated with the following GO terms: “DNA replication-dependent nucleosome assembly,” “chromatin silencing at rDNA,” “CENP-A-containing nucleosome assembly,” and “DNA replication-independent nucleosome organization.” Conversely, hypomethylated genes in brain metastases demonstrated enrichment in pathways related to “gene silencing by miRNA,” “posttranscriptional gene silencing,” and “gene silencing by RNA” (Supplementary Table S3).

## Discussion

Breast cancer is a heterogeneous disease clinically classified by ER, PR, and HER2 status. TNBC and HER2-positive subtypes exhibit aggressive progression and a high incidence of brain metastases, posing substantial therapeutic challenges despite advances in systemic treatment. Epigenetic dysregulation, particularly via DNA methylation, can silence tumor suppressors or activate oncogenes without altering DNA sequences, contributing to carcinogenesis 26, 27. These changes may also affect microRNA regulation, impacting apoptosis and proliferation.

In this study, we analyzed BCBM and primary BC samples using the Illumina Infinium Methylation EPIC Bead Chip. A total of 107 CpG sites showed nominal differential methylation (Δβ ≥ 0.1; p-value < 0.05), including 97 promoter and 10 gene regions. The top altered genes and promoters included *HMGB1P7*, *DEFB1*, *MIR5089*, *NKX2-6*, *OR51A10P*, *LINC00528*, *SLC15A3*, *PCDHGB7*, *MIR4708*, and *RNU6-993P*.

Having a closer look at the top ten differentially methylated genes, we found strong correlation being associated with tumorigenesis. *DEFB1* supports antitumor immunity by activating adaptive responses, and shows tumor-suppressive function 28-31. *HMGB1P7H*, although annotated as a pseudogene, may regulate gene expression similarly to microRNAs and lncRNAs 32, 33 and participate in maintaining genomic stability, activating oncogenic pathways and promoting tumor progression, therapy resistance, and immune evasion 34-44. Olfactory-receptor pseudogene *OR51A10P* may also influence cancer biology by implicating in tumor signaling and biomarker development 45-48. Two microRNAs showed altered methylation in BCBM: *MIR5089* (hypomethylated) and *MIR4708* (hypermethylated). MicroRNAs broadly regulate post-transcriptional gene expression and can function as oncogenes or suppressors 49, 50. *MIR5089*-5p has been proposed as a circulating biomarker in breast cancer 51, while *MIR4708* may contribute to TNBC risk stratification 52. *LINC00528*, a hypermethylated lncRNA, likely acts as a tumor suppressor by modulating immune and adhesion pathways. Its reduced expression correlates with advanced HER2-positive BC and poor survival outcomes 53. *RNU6-993P*, another pseudogene, lacks direct cancer evidence, though related U6 family members have diagnostic relevance 54, 55. *NKX2-6* encodes a transcription factor involved in neural developmental pathways 56, and related *NKX2 genes* can promote chemoresistance in breast cancer 57. *SLC15A3* (synonym *PHT2*), an immune-regulatory solute transporter, and other *SLC* family members have emerging roles as cancer biomarkers 59-62. *PCDHGB7* promotes apoptosis and cell-cycle arrest and increases TNBC sensitivity to carboplatin, supporting its utility as a serum biomarker 63, 64. Though all of these genes have been reported to have a role in other cancer types, their relevance to brain metastasis requires further investigation.

A major limitation of this study is the absence of matched transcriptomic data; therefore, whether the identified differentially methylated sites affect gene expression is unknown. To support the biological relevance of our findings we performed validation of our dataset by searching in online databases. We found the three genes *LINC00528*, *SLC15A3* and *PCDHGB7* in an extended TCGA-BRCA (The Cancer Genome Atlas Breast Cancer) dataset with additional histologic type annotations in a total of 1,063 breast cancers. The genes are associated with a special gene expression signature containing significant upregulated genes associated with rare subtypes 65.

Prior studies highlight hypermethylated promoters as potential markers for BC progression and brain metastasis 66-67. In our cohort, 107 CpG sites (Δβ ≥ 0.1; p < 0.05) were nominally significantly methylated between BCBM and primary BC tissues. Our epigenomic analysis revealed that, on a global scale, metastatic brain lesions exhibit greater similarity to their respective primary breast tumors than the primary tumors do to each other (Figure 2A, B). This observation underscores the clonal and lineage-dependent nature of metastatic progression, suggesting that key molecular features are preserved during dissemination. Matched primary and metastatic samples clustered together, reflecting patient-specific epigenetic patterns, but this does not preclude the existence of smaller, biologically relevant methylation changes associated with metastatic progression.

Despite this overarching resemblance, the data also highlight a subset of genes with distinct methylation and expression patterns that appear specific to the metastatic process itself. These loci may contribute to the acquisition of traits essential for successful colonization of distant organs, such as enhanced migratory capacity, immune evasion, or adaptation to the brain microenvironment. Notably, differential methylation was detected in several non-coding RNAs and pseudogenes, pointing to epigenetic reprogramming as a driver of metastatic competency. These findings suggest a dual landscape: while metastases largely recapitulate their tumor of origin, specific epigenetic alterations demarcate them as biologically distinct entities. This distinction may hold translational relevance for biomarker development and therapeutic targeting, particularly in the context of brain metastases, which remain a clinical challenge with limited treatment options.

A limitation of this study is on the one hand the small sample size of only ten matched cases and then that cohort includes tumors of all major breast cancer subtypes without stratification by ER, PR, or HER2 status. Because intrinsic subtypes display distinct baseline methylation landscapes, this heterogeneity may impede the detection of subtype-specific metastasis-associated alterations or diminish signals driven by particular biological contexts. Our findings nonetheless provide valuable preliminary insights into epigenetic alterations associated with brain metastasis in breast cancer. The restricted cohort size may constrain the generalizability of the results and preclude robust statistical power; however, the paired design and focus on methylation profiling across primary and metastatic sites offer a unique perspective that is rarely captured in larger datasets.

Future studies with larger, subtype-balanced cohorts will be essential to determine which components of the signature are universal versus subtype-restricted. These initial observations lay the groundwork for future investigations and underscore the importance of integrating epigenomic data into the study of metastatic progression. Furthermore, a major limitation of this study is the lack of direct *in vitro* functional assays. This must be clarified in further research. Despite its exploratory nature, the study contributes meaningfully to a growing body of literature and warrants dissemination to stimulate further research in this domain.

## Supplementary Material

Supplementary tables.

## Figures and Tables

**Figure 1:**
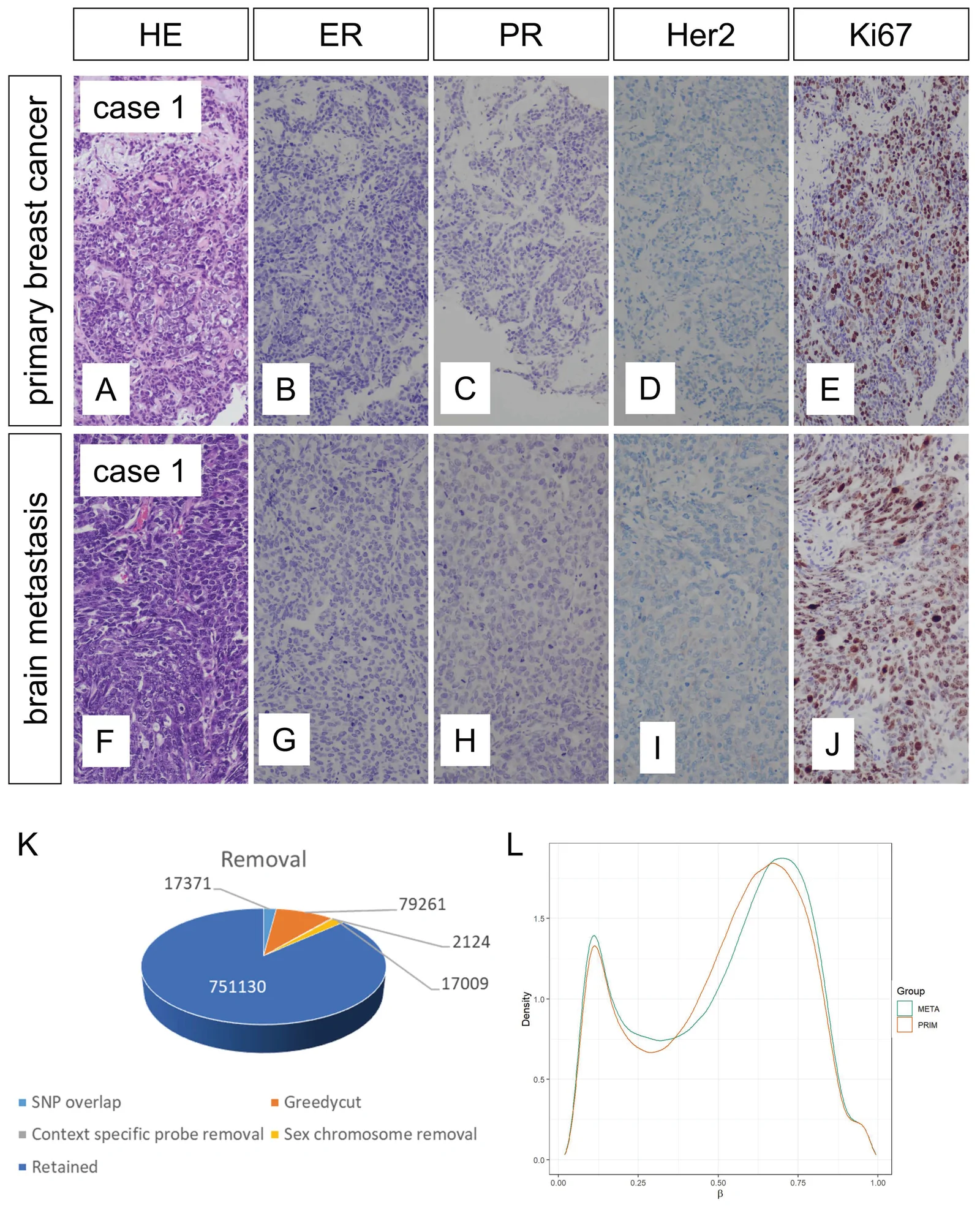
Primary breast cancer and brain metastasis. Exemplified pictures of primary breast cancer (A-E) and brain metastasis (F-J) (Case 1). During preprocessing of the 866,895 probes 17,371 probes were removed that overlap with SNPs, 79,261 applying greedycut filtering, 2,124 context specific probes and 17,009 probes on sex-chromosomes resulting in a total of 751,130 retained probes (K). Global beta value distributions showed high densities with beta values <0.1 and >0.8 (L). HE: hemalaun-eosin stain; ER: estrogen receptor; PR: progesterone receptor; magnification: 20x.

**Figure 2:**
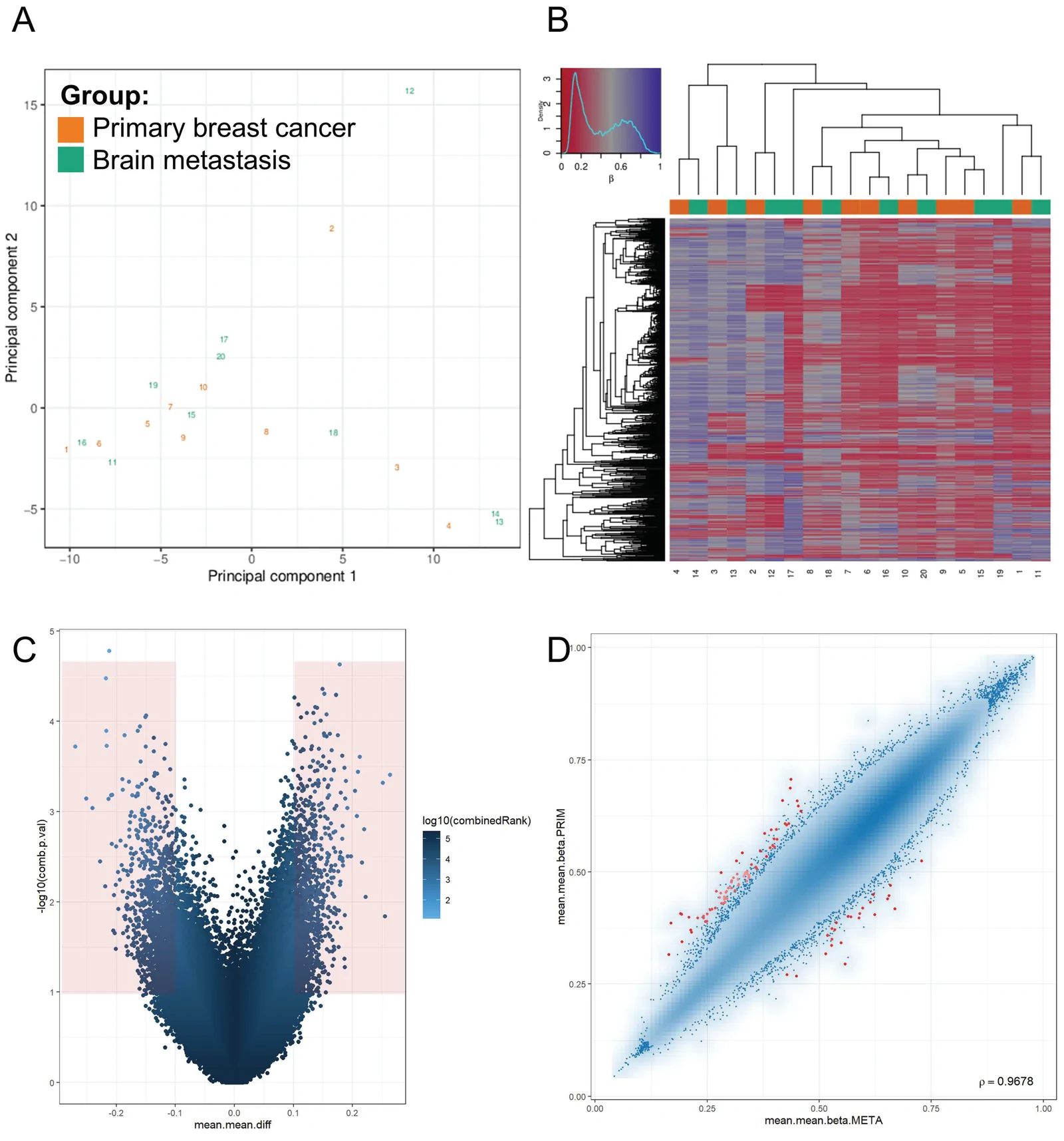
Methylation analysis. Principal component analysis (PCA) revealed that primary breast cancer and brain metastases do not form two distinct clusters. Instead, samples from individual patients, namely the primary tumor and its corresponding brain metastasis, were located in close proximity, suggesting a conserved epigenetic relationship between the two tissue types (pairs of primary cancer and metastasis: 1/11; 2/12; 3/13; 4/14; 5/15; 6/16; 7/17; 8/18; 9/19; 10/20.) (A). Clustering based on unsupervised hierarchical methods and random forest analysis further demonstrated the absence of two distinct groups. Instead, primary breast cancer and its corresponding brain metastases appeared in close proximity when considering the 1,000 most variable CpG sites, suggesting a shared epigenetic landscape between the matched tumor samples (B). Volcano plot shows nominally significantly methylated sites (Δβ ≥0.1, p <0.05) indicated in red (C). Scatter plot shows the combined rank highlighting the top 100 best ranking regions in red (D).

**Figure 3:**
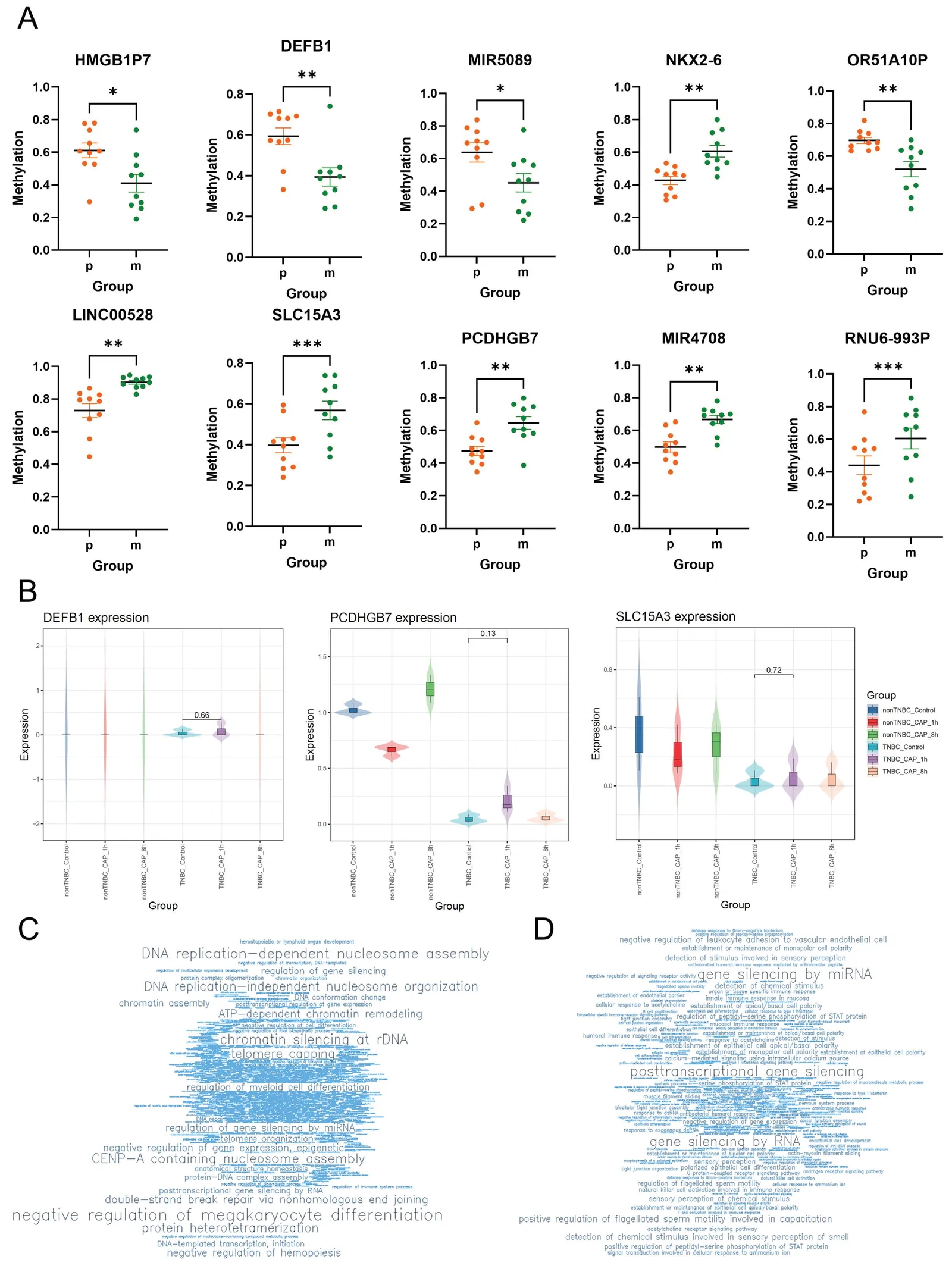
Differential methylation analysis. Methylation levels of top 10 differentially methylated regions show significant differences in primary breast cancer compared with brain metastasis (A) (t-test; indicated are mean and SEM); p: primary breast cancer; m: brain metastasis. Transcriptomic screening of core differentially methylated loci under redox perturbation using CAPmed-BC (B). Violin plots display the expression levels of *DEFB1*, *PCDHGB7*, and *SLC15A3* across non-TNBC and TNBC breast cancer models. Experimental groups include untreated controls (Control) and cells exposed to cold atmospheric plasma (CAP) after 1 hour (CAP_1h) and 8 hours (CAP_8h). Gene ontology (GO) enrichment analysis showed the top 1000 best ranking genes by word clouds of top terms being enriched in hypomethylated (C) and hypermethylated (D) pathways. * p < 0.05; ** p < 0.01; *** p < 0.001.

## Data Availability

The datasets generated and analyzed during the current study are not publicly available in open repositories due to legal and ethical restrictions under the European General Data Protection Regulation (GDPR), as they contain sensitive human biometric data. However, the data are available for independent scientific verification from the corresponding author upon completion of a formal Data Transfer Agreement (DTA) and approval by our institutional data protection compliance office. Interested researchers may initiate this process by contacting the corresponding authors.
