# Supplementary material for: Epigenome-Wide DNA Methylation Profiling of Matched Primary Breast Cancers and Brain Metastases Reveals Epigenetic Markers linked to Metastatic Progression: A Preliminary Study
Source: Int J Med Sci. 2026 Oct 2;23(11):3554–64. doi: 10.7150/ijms.130407 (PMC13685463; doi:10.7150/ijms.130407)
Supplement: Supplementary file 1 [file ijmsv23p3554s1.pdf]

Table S1: Patient characteristics. IHC = immunohistochemical, HR = hormone receptor, HER2 = human epidermal growth factor receptor 2, BM = brain metastasis, P = primary tumor, TNBC = triple-negative breast cancer, NST = no special type. * Local recurrence; ** Her2 2+ ISH ratio 1.6; *** bilateral breast cancer.

Pat-ID primary/metastasis	Age at BM surgery	Tissue	IHC subtype	Grade	Histology	Tumor cell content	Barcode/ Sentrix_ID	Run date
1/11	58	P	TNBC	3	NST	75%	205676380114_R04C01	15.12.2021
		BM	TNBC	3	NST + lobular	95%	205676380114_R05C01	15.12.2021
2/12	37	P*	HR+/HER2+	3	NST	70%	205676380114_R06C01	15.12.2021
		BM	HR-/HER2+	3	NST	80%	205676380114_R07C01	15.12.2021
3/13	45	P	HR+/HER2+	2	NST	60%	205676380114_R08C01	15.12.2021
		BM	HR+/HER2+	3	NST	85%	205676380164_R01C01	15.12.2021
4/14	62	P	HR+/HER2-	2	lobular	80%	205676380164_R03C01	15.12.2021
		BM	HR+/HER2-	3	lobular	90%	205676380164_R04C01	15.12.2021
5/15	42	P	HR+/HER2-	2	NST	65%	205676380164_R05C01	15.12.2021
		BM	HR+/HER2-	2	NST	90%	205676380164_R06C01	15.12.2021
6/16	58	P*	TNBC	3	NST	60%	205676380164_R07C01	15.12.2021
		BM	TNBC	3	NST	50%	205676380164_R08C01	15.12.2021
7/17	57	P	TNBC	2	NST	50%	205724790007_R03C01	22.12.2021
		BM	HR+/HER2-**	3	NST	90%	205724790007_R04C01	22.12.2021
8/18	52	P	HR+/HER2-	3	NST	50%	205724790007_R05C01	22.12.2021
		BM	HR+/HER2-	3	NST	80%	205724790007_R06C01	22.12.2021
9/19	49	P***	HR+/HER2-	3	NST	30%	205724790007_R07C01	22.12.2021
		BM	HR-/HER2+	3	NST	70%	205724780073_R01C01	22.12.2021
10/20	38	P	HR+/HER2-	3	NST	70%	205724780073_R02C01	22.12.2021
		BM	HR+/HER2+	3	NST	65%	205724780073_R03C01	22.12.2021

Table S2. Differentially methylated CpGs. List of ten DMCGs shows two in gene and eight in promoter regions (Δβ ≥0.1, p <0.05).

ID	Chr	symbol	mean.mean.META	mean.mean.PRIM	mean.mean.diff	abs.mean.mean.dif	comb.p.val	combinedRank	num.sites	location
ENSG00000231148	chr10	HMGB1P7	0.41	0.61	-0.20	0.20	0.01	55	1	promoter
ENSG00000164825	chr8	DEFB1	0.39	0.59	-0.20	0.20	0.00	17	1	gene
ENSG00000264999	chr17	MIR5089	0.45	0.64	-0.19	0.19	0.02	231	1	promoter
ENSG00000180053	chr8	NKX2-6	0.61	0.43	0.18	0.18	0.01	108	15	promoter
ENSG00000230484	chr11	OR51A10P	0.52	0.70	-0.18	0.18	0.00	2	1	promoter
ENSG00000269220	chr22	LINC00528	0.90	0.73	0.17	0.17	0.00	317	1	gene
ENSG00000110446	chr11	SLC15A3	0.57	0.40	0.17	0.17	0.01	57	5	promoter
ENSG00000254122	chr5	PCDHGB7	0.65	0.47	0.17	0.17	0.00	62	10	promoter
ENSG00000266740	chr14	MIR4708	0.67	0.50	0.17	0.17	0.00	133	1	promoter
ENSG00000252962	chr19	RNU6-993P	0.60	0.44	0.16	0.16	0.05	597	1	promoter

Table S3. Gene ontology enrichment. Analysis shows enrichment of pathways being associated "DNA replication-dependent nucleosome assembly", "chromatin silencing at rDNA", "CENP-A containing nucleosome assembly", and "DNA replication-independent nucleosome organization" within to ten pathways being hypermethylated and "gene silencing by miRNA", "posttranscriptional gene silencing", and "gene silencing by RNA" as top terms being hypomethylated in BM compared with primary BC.

Enrichment analysis of brain metastasis compared with primary cancer: hypermethylated

GOMFID	Pvalue	OddsRatio	ExpCount	Count	Size	Term
GO:0045653	0	2,074	0.05	14	18	negative regulation of megakaryocyte differentiation
GO:0006335	0	460	0.08	14	32	DNA replication-dependent nucleosome assembly
GO:0000183	0	345	0.10	14	38	chromatin silencing at rDNA
GO:0034080	0	296	0.11	14	42	CENP-A containing nucleosome assembly
GO:0034724	0	218	0.13	14	52	DNA replication-independent nucleosome organization
GO:0051290	0	207	0.14	14	54	protein heterotetramerization
GO:0016233	0	202	0.14	14	55	telomere capping
GO:0006303	0	122	0.21	14	82	double-strand break repair via nonhomologous end joining
GO:0043044	0	116	0.21	14	85	ATP-dependent chromatin remodeling
GO:1903707	0	70	0.37	15	146	negative regulation of hemopoiesis

Enrichment analysis of brain metastasis compared with primary cancer: hypomethylated

GOMFID	Pvalue	OddsRatio	ExpCount	Count	Size	Term
GO:0035195	1.00E-04	13.8746	0.48	5	422	gene silencing by miRNA
GO:0016441	1.00E-04	13.5088	0.49	5	433	posttranscriptional gene silencing
GO:0031047	1.00E-04	12.7148	0.52	5	459	gene silencing by RNA
GO:0060474	0.0034	461.5278	0.00	1	3	positive regulation of flagellated sperm motility involved in capacitation
GO:0050911	0.0057	9.4885	0.37	3	325	detection of chemical stimulus involved in sensory perception of smell
GO:1904995	0.0068	184.5778	0.01	1	6	negative regulation of leukocyte adhesion to vascular endothelial cell
